# Evaluation of the plaque removal efficacy of chewable toothpaste tablets – a randomized controlled crossover trial

**DOI:** 10.3389/froh.2026.1893652

**Published:** 2026-08-21

**Authors:** Harshit Srivastava, Kumar Gaurav Chhabra, Naganandini S., Pankaj Chaudhary, Shweta Dangi, Nimisha Srivastava

**Affiliations:** 1 Department of Public Health Dentistry, Nims Dental College and Hospital, Nims University Rajasthan, Jaipur, India; 2 Department of Oral Medicine and Radiology, Nims Dental College and Hospital, Nims University Rajasthan, Jaipur, India

**Keywords:** chewable toothpaste tablets, herbal dentifrice, plaque control, preventive dentistry, randomized crossover trial, sustainable oral healthcare

## Abstract

**Background:**

Chewable toothpaste tablets (CTTs) have recently emerged as environmentally sustainable alternatives to conventional toothpaste formulations. However, limited scientific evidence is available regarding their clinical efficacy in plaque control. The present study aimed to compare the antiplaque efficacy and user acceptability of two commercially available chewable toothpaste tablets among young adults.

**Materials and methods:**

A randomized double-blind crossover clinical trial was conducted among 196 adults aged 18–35 years. Participants were randomly allocated into two intervention sequence groups (AB and BA). Sequence AB used Apollo Noni CTT for 7 days followed by Teeth-A-Bit after a 7-day washout period, whereas Sequence BA followed the reverse sequence. Plaque scores were recorded using the Modified Turesky–Gilmore–Glickman Plaque Index following 24-h abstinence from oral hygiene procedures at baseline and after each intervention phase. Participant feedback was assessed using a structured questionnaire. Data were analyzed using descriptive statistics, chi-square tests, and linear mixed-effects modeling with restricted maximum likelihood estimation.

**Results:**

A total of 196 participants (95 men, 101 women; mean age 23.42 ± 2.65 years) completed the study. Baseline plaque scores were comparable between the AB and BA treatment sequences (Period 1: 1.82 ± 0.24 vs. 1.77 ± 0.25; Period 2: 1.77 ± 0.26 vs. 1.80 ± 0.19). Following the 7-day intervention, plaque scores decreased in both sequences, with lower post-intervention scores observed for Apollo Noni (1.34 ± 0.27 and 1.32 ± 0.21) than for Teeth-A-Bit (1.54 ± 0.24 and 1.52 ± 0.27). Linear mixed-effects analysis showed baseline plaque score significantly predicted post-treatment score (*p* < 0.001). After adjustment, Apollo Noni achieved significantly lower plaque scores than Teeth-A-Bit (1.322 ± 0.010 vs. 1.548 ± 0.010; mean difference −0.226, 95% CI: −0.245 to −0.206; *p* < 0.001). No significant period or sequence effects were found, indicating no carryover bias. Both products were well-accepted, with no adverse effects or significant gender-based differences in user experience or satisfaction (*p* > 0.05).

**Conclusion:**

Both CTTs reduced dental plaque, but Apollo Noni demonstrated noticeably greater antiplaque efficacy. The results indicate that CTTs may offer viable, eco-friendly substitutes for traditional dentifrices.

**Clinical Trial Registration:**

https://ctri.nic.in/Clinicaltrials/pmaindet2.php?EncHid=MTEwNTg2&Enc=&userName=, Clinical Trials Registry–India (CTRI/2024/07/070865).

## Introduction

Oral diseases continue to place a significant burden on public health systems and are one of the most common chronic ailments worldwide. Billions of people suffer from dental caries and periodontal disorders, which have a substantial negative impact on systemic health outcomes, nutritional status, and quality of life (1). These illnesses are nevertheless very common in both affluent and developing nations, even though they are mostly preventable.

Dental plaque is a structurally ordered microbial biofilm contained in an extracellular polymeric matrix that sticks to tooth surfaces. The metabolic processes of local microorganisms, which cause gingival inflammation and enamel demineralization, are principally responsible for the pathogenic potential of plaque biofilm (2).

The role of plaque biofilm in the initiation of periodontal disease was confirmed by the first experimental gingivitis study conducted by Loe et al., in which a direct causal link was established between the accumulation of plaque biofilm and inflammation of the gums (3). Furthermore, the current evidence suggests that dysbiotic changes in the oral biofilm environment are an important step in the pathogenesis of periodontitis from gingivitis (4). For these reasons, the maintenance of a good plaque-removal routine remains the mainstay of periodontal therapy and preventative dental care. The most universally used method for removing plaque is regular brushing of teeth. This brushing regimen has been shown to significantly decrease plaque and improve gingival health outcomes in comprehensive evaluations (5). Fluoride and antimicrobial-containing dentifrices reduce the microbial population in the mouth and enhance the effectiveness of oral hygiene through remineralization, in addition to the mechanical cleaning action (6, 7). Environmental sustainability has increasingly been gaining significance in the consumer product and healthcare sectors in the past few years. One of the main sources of plastic waste in the environment is the multi-layer plastic laminate tubes that contain traditional toothpaste, which are not easily recyclable (8–10). This has created an increased awareness of sustainability and plastic waste, leading to increased interest in eco-friendly dental care options. Chewable toothpaste tablets (CTTs) are compressed dentifrices without water that are chewed before brushing with a wet toothbrush. These products help to reduce reliance on the standard plastic toothpaste tube as they are frequently sold in reusable or recyclable containers. Due to the absence of water, these products are more stable and reduce the need for preservatives. The effectiveness of herbal and alternative dentifrices in removing plaque and enhancing gingival health has been tested in a number of studies (11–19). The use of short-term plaque regrowth models and a small carryover effect when appropriate washout periods are provided were demonstrated by previous crossover dentifrice studies (12, 20, 21). Moreover, the antibacterial and antiplaque effects of the following herbal products have been shown to be effective in plaque reduction: *Salvadora persica* (miswak), clove, cinnamon, tea tree oil, and xylitol (13–16, 22–27). Recent studies on chewable toothpaste tablets have found positive plaque-removal results and environmental benefits (28–33). Nevertheless, there is still a dearth of direct comparative data assessing different chewable toothpaste tablet compositions.

Apollo Noni and Teeth-A-Bit were selected in this study because they are commercially available chewable toothpaste tablets that were readily accessible during the study period and represent products currently available to consumers. Apollo Noni is an established commercial brand with a herbal formulation, whereas Teeth-A-Bit is a fluoride-containing chewable toothpaste tablet with a different ingredient profile. Although other chewable toothpaste tablets are available, many share similar formulations, and no previous randomized clinical trial has directly compared these two commercially available products under standardized conditions. Importantly, neither manufacturer provided financial support or study materials, nor did either have any role in the study design, data collection, analysis, interpretation, or manuscript preparation. Product selection was made independently by the investigators.

Hence, the present study aimed to assess and compare the plaque removal effectiveness of two chewable toothpaste tablets, Apollo Noni and Teeth-A-Bit, using the Modified Turesky–Gilmore–Glickman Plaque Index. Secondary outcomes were the acceptability of the products, including the overall experience, satisfaction, comfort while using the product, and willingness to continue using the products, and the occurrence of adverse events related to the use of the products.

## Materials and methods

### Study design and ethical approval

A randomized double-blind crossover clinical trial was conducted in the Department of Public Health Dentistry, Nims University Rajasthan, Jaipur, India, over a period of 12 months. Ethical approval was obtained from the Institutional Ethics Committee of Nims University (Approval No. IEC/P535-/2024). The study protocol was prospectively registered with the Clinical Trials Registry–India (CTRI/2024/07/070865).

The study was conducted in accordance with the Declaration of Helsinki and the Consolidated Standards of Reporting Trials (CONSORT) 2010 extension guidelines for randomized crossover trials (34, 35).

### Study population

The study population comprised systemically healthy young adults aged between 18 and 35 years attending the Department of Public Health Dentistry.

### Inclusion criteria

- Systemically healthy individuals aged 18–35 years.
- Participants willing to provide written informed consent.
- Participants willing to use chewable toothpaste tablets during the study period.
- Participants willing to abstain from oral hygiene procedures for 24 h before clinical examinations.
- Participants willing to discontinue existing dentifrice products and comply with study instructions.

### Exclusion criteria

- Antibiotic use within the previous 15 days.
- Presence of extensive untreated dental caries or oral mucosal diseases.
- Participants wearing orthodontic appliances.
- Participants with abnormal salivary flow.
- Individuals with multiple missing teeth or established periodontitis.

### Sample size calculation

The sample size was estimated using G*Power version 3.1.9.4. The expected effect size Cohen's *d* = 0.27 was chosen because it is in line with the magnitude of plaque-regrowth inhibition reported in previous dentifrice studies and is based on the results of a systematic review and meta-analysis by Valkenburg et al. (36), which showed that dentifrices generally inhibit the regrowth of plaque by approximately 0.25 or more than control interventions. Thus, a conservative effect size estimate of the expected clinically meaningful difference for sample size determination was 0.27, as the expected treatment differences in the plaque regrowth studies were generally small to moderate. Based on a two-sided alpha (*α*) value of 0.05 and 95% (1−*β*) power, a sample size of 196 participants was required, after accounting for attrition. Thus, 98 patients were required in each group, which were crossed over to another group. The recruitment period of the patients in this clinical trial was 25/07/2024 to 25/07/2025.

### Randomization and allocation concealment

Participants were screened for selection based on the inclusion and exclusion criteria, medical history, and clinical oral examinations for conditions associated with reduced salivary flow. Individuals with clinically evident signs of oral dryness, a history of xerostomia or salivary gland disorders, or the use of medications known to affect salivary secretion were excluded from the study. The participants were randomly allocated into two intervention sequence groups (AB and BA) using computer-generated block randomization with a block size of four generated by an independent investigator not involved in data collection or outcome assessment. Allocation concealment was ensured using sequentially numbered opaque sealed envelopes.

### Blinding

Both participants and outcome assessors remained blinded throughout the study period. The chewable toothpaste tablet formulations were dispensed in identical coded containers prepared by an independent investigator to maintain double blinding.

### Preparatory training phase

A preparatory training phase of 1 week was conducted, during which all participants received standardized oral hygiene instructions and demonstration of the Modified Bass toothbrushing technique. Standardized soft-bristle toothbrushes were provided to all participants.

### Intervention protocol

Participants were assigned to one of the following intervention sequences: Sequence AB (*n* = 98): Apollo Noni for 7 days followed by Teeth-A-Bit after washout; or Sequence BA (*n* = 98): Teeth-A-Bit for 7 days followed by Apollo Noni after washout.

The first intervention phase lasted for 7 days. Baseline plaque scores were recorded following 24-h abstinence from oral hygiene procedures. Participants were instructed to brush twice daily using one chewable toothpaste tablet per brushing session.

**Figure 1:**
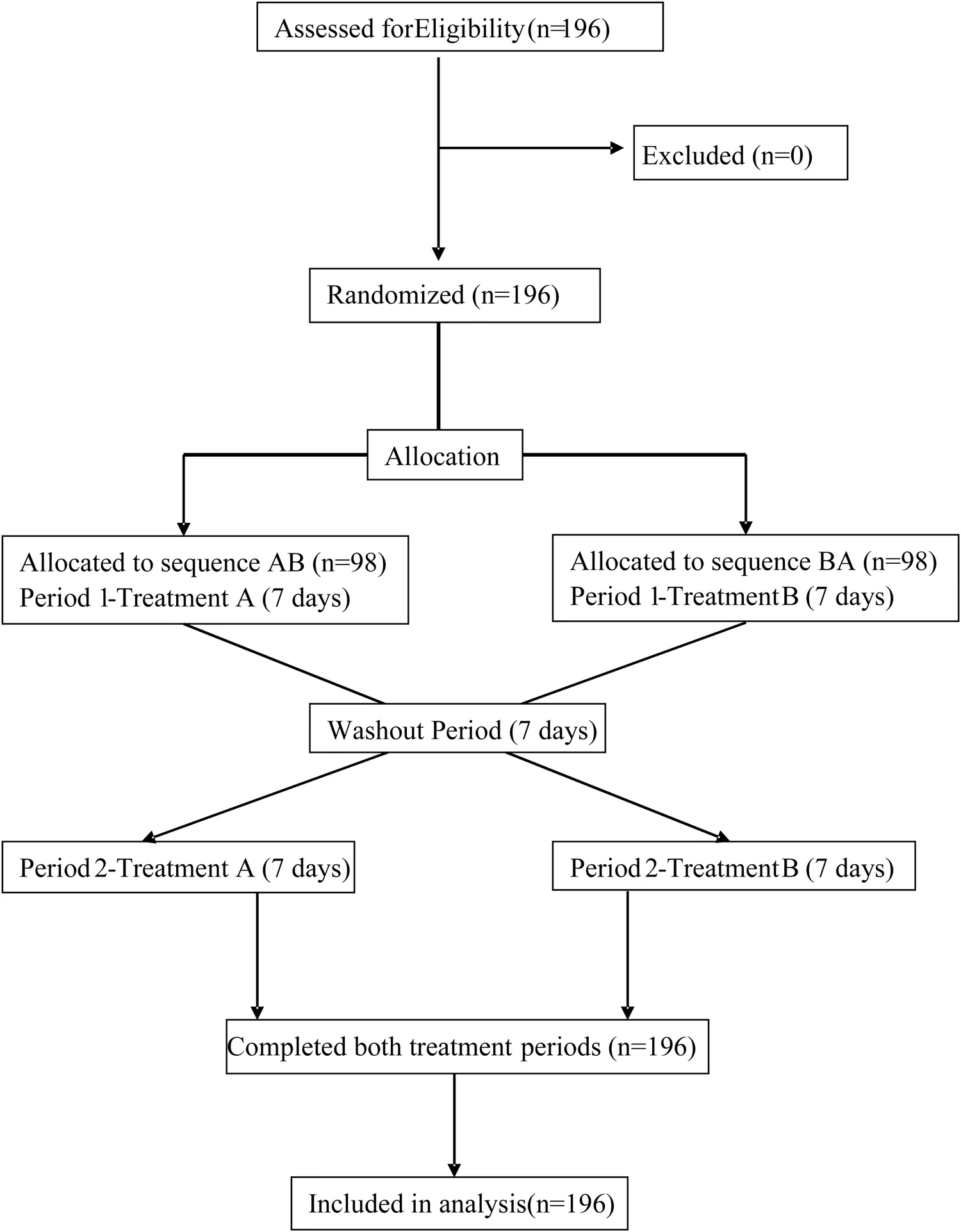
CONSORT chart of the study.

Following completion of Phase I, participants underwent a 7-day washout period during which they resumed their routine oral hygiene practices. Subsequently, baseline plaque scores for Phase II were recorded after 24-h abstinence from oral hygiene measures, followed by crossover to the alternate intervention for another 7-day period (Figure 1).

Daily reminder messages were sent during each intervention period and the participants were asked to return the tablet container during their follow-up visits. These procedures were adopted to encourage and track compliance with the study protocol.

Each follow-up visit was used to ask the participants about the presence of any adverse events such as oral irritation, burning sensation, ulceration, discomfort, allergic reactions, or any other unusual oral symptoms that may have occurred during the intervention period. The investigator also examined the oral cavity clinically at each of the assessment visits. No adverse events were reported or observed in either intervention period.

The 7-day washout period employed in this study was based on previously published plaque regrowth and crossover dentifrice investigations demonstrating adequate elimination of residual treatment effects with substantially shorter washout intervals ranging from 4 days to 7 days (12, 20, 21, 37). Addy et al. showed that the use of short-term plaque regrowth models was valid and implemented sufficient washout periods to bring plaque accumulation patterns back to normal levels between the treatment phases (12). Likewise, Claydon et al. used a 7-day washout in crossover toothpaste research and proved its methodological adequacy, with no substantial carryover effects (22). Hence, the washout period used in this study was considered appropriate.

### Study products

#### Teeth-A-Bit chewable toothpaste tablet

The commercially available Teeth-A-Bit chewable toothpaste tablet formulation contained menthol (*Mentha arvensis*), peppermint powder (*Mentha piperita*), clove powder (*Syzygium aromaticum*), xylitol, calcium carbonate, natural salt (sodium chloride), and sodium monofluorophosphate containing approximately 1,000 ppm fluoride.

#### Apollo Noni chewable toothpaste tablet

The Apollo Noni chewable toothpaste tablet formulation contained clove, cinnamon, meswak (*S. persica*), natural plant extracts, natural menthol, non-GMO xylitol, non-GMO mannitol, and sweeteners.

### Outcome assessment

After applying a disclosing solution, plaque accumulation was measured using the Modified Turesky–Gilmore–Glickman Plaque Index.

The Modified Turesky–Gilmore–Glickman Plaque Index was used for examiner calibration prior to the start of the trial. Significant agreement was shown in the intra-examiner reliability test (kappa = 0.86).

At the conclusion of Phase II, a structured study-specific questionnaire consisting of five closed-ended items was used to evaluate the participants' overall experience with the chewable toothpaste tablets, their satisfaction with their use compared with conventional toothpaste, their willingness to continue using the product, their comfort with long-term use, and the occurrence of any self-reported adverse effects. Overall experience was rated on a five-point ordinal scale (excellent, very good, good, bad, and very bad), while satisfaction was assessed using a 10-point numerical rating scale (1 = unsatisfied; 10 = highly satisfied). The remaining items were recorded as dichotomous (yes/no) responses. As the questionnaire was designed for exploratory clinical assessment rather than formal psychometric evaluation, validation and reliability testing were not performed.

### Statistical analysis

Data analyses were performed using IBM SPSS Statistics version 27.0. Normality testing was conducted using the Kolmogorov–Smirnov test. Descriptive statistics are expressed as mean ± standard deviation.

A linear mixed-effects model using restricted maximum likelihood (REML) estimation with Satterthwaite approximation was employed to evaluate the treatment, period, sequence, and treatment × period interaction effects associated with the crossover design. Fixed variables included treatment, period, sequence, and treatment × period interaction, while participant ID was treated as a random intercept to account for within-subject correlation across crossover periods. An unstructured covariance matrix was specified.

For categorical variables, the chi-square test was employed. Multiple comparisons were not adjusted for since the participant feedback analyses were exploratory in nature. Statistical significance was determined at *p* < 0.05

## Results

A total of 196 participants were enrolled in the study, comprising 95 men (48.5%) and 101 women (51.5%). The overall mean age of the study population was 23.42 ± 2.65 years. The mean age of the male participants was 23.81 ± 2.59 years, while that of the female participants was 23.05 ± 2.67 years. Overall, the study population was demographically comparable with respect to age and gender (Table 1).

**Table 1:** Demographic characteristics of the study participants.

Variable	Male (*n* = 95)	Female (*n* = 101)	Total (*N* = 196)
Age (years) (M ± SD)	23.81 ± 2.59	23.05 ± 2.67	23.42 ± 2.65
Gender *n* (%)	95 (48.5%)	101 (51.5%)	196 (100%)

The descriptive analysis of the plaque scores according to treatment sequence and study period showed that in the AB sequence, mean plaque scores decreased from 1.82 ± 0.24 to 1.34 ± 0.27 in Period 1 and from 1.77 ± 0.26 to 1.52 ± 0.27 in Period 2. Similarly, in the BA sequence, mean plaque scores decreased from 1.77 ± 0.25 to 1.54 ± 0.24 in Period 1 and from 1.80 ± 0.19 to 1.32 ± 0.21 in Period 2 (Table 2). However, an inferential statistical analysis using the linear mixed-effects model is presented separately to account for treatment, period, and sequence effects inherent to the crossover design.

**Table 2:** Baseline and post-intervention plaque scores according to treatment sequence and study period.

Sequence	Outcome	Period 1 (mean ± SD)	Period 2 (mean ± SD)
AB	Baseline plaque	1.82 ± 0.24	1.77 ± 0.26
	Post-intervention plaque	1.34 ± 0.27	1.52 ± 0.27
BA	Baseline plaque	1.77 ± 0.25	1.80 ± 0.19
	Post-intervention plaque	1.54 ± 0.24	1.32 ± 0.21

**Table 3:** Linear mixed-effects model results for plaque score.

Fixed effect	F (df1, df2)	*p*-value
Baseline score	752.25 (1, 349.74)	<0.001
Treatment	510.66 (1, 168.64)	<0.001
Period	0.21 (1, 167.56)	0.645
Sequence	0.27 (1, 167.74)	0.608
Variance components		
Parameter	Estimate	Standard error
Subject variance (random intercept)	0.0105	0.0018
Residual variance	0.0097	0.0011

A linear mixed-effects model was fitted using REML, with baseline plaque score as a covariate and subject as a random intercept. Denominator degrees of freedom were estimated using the Satterthwaite approximation.

**Table 4:** Adjusted treatment effects and pairwise comparison from the linear mixed-effects model.

Treatment	Adjusted mean (SE)	95% confidence interval	
Apollo Noni	1.322 (0.010)	1.302–1.342	
Teeth-A-Bit	1.548 (0.010)	1.528–1.568	
Pairwise comparison of treatments			
Comparison	Mean difference (SE)	95% confidence interval	*p*-value
Apollo Noni vs. Teeth-A-Bit	−0.226 (0.010)	−0.245 to −0.206	<0.001

Estimated marginal means were adjusted for the mean baseline plaque score (baseline score = 1.7954). Pairwise comparisons were performed using Bonferroni adjustment.

Baseline plaque score was a highly significant predictor of post-treatment plaque score [F (1,349.74) = 752.25, *p* < 0.001]. After adjusting for baseline plaque levels, a significant treatment effect was observed [F (1,168.64) = 510.66, *p* < 0.001], indicating that plaque scores differed significantly between the two toothpaste tablet formulations. Neither the period effect [F (1,167.56) = 0.21, *p* = 0.645] nor the sequence effect [F (1,167.74) = 0.27, *p* = 0.608] reached statistical significance, suggesting the absence of period-related bias and carryover effects. Variance component estimates demonstrated appreciable between-subject variability (*σ*^2^ = 0.0105) and residual variability (*σ*^2^ = 0.0097), supporting the inclusion of a random intercept in the model (Table 3).

The adjusted mean plaque score was significantly lower for the Apollo Noni toothpaste tablet (1.322 ± 0.010) than for the Teeth-A-Bit toothpaste tablet (1.548 ± 0.010). A pairwise comparison demonstrated a statistically significant adjusted mean difference of −0.226 units (95% CI: −0.245 to −0.206; *p* < 0.001), indicating the superior antiplaque efficacy of Apollo Noni. The confidence interval excluded zero, confirming the robustness and precision of the treatment effect after controlling for baseline plaque scores and accounting for within-subject correlation in the crossover design (Table 4).

The Pearson chi-square test was used to assess the relationship between participant feedback factors and gender. No statistically significant correlation was observed between gender and overall experience with chewable tablets (*χ*^2^ = 2.369, df = 2, *p* = 0.306). Men reported an “excellent” experience in 11.6% of cases, while women reported an “excellent” experience in 12.9% of cases. Nonetheless, the majority of participants in both groups gave their experience a “very good” rating (54.7% for men and 63.4% for women). There was no statistically significant correlation between gender and satisfaction level (*χ*^2^ = 0.083, df = 1, *p* = 0.773). The lack of a significant correlation was further confirmed by Fisher's exact test (*p* = 0.881), indicating that the men and women had similar levels of satisfaction. None of the participants reported any negative side effects during the course of the study. No chi-square statistic was calculated because the variable remained constant between the groups. Similarly, there was no significant gender difference in the willingness to continue using chewable tablets (*χ*^2^ = 0.053, df = 1, *p* = 0.818; Fisher's exact *p* = 0.865). The percentage of women who said they would be willing to continue was slightly greater (78.2%) than that of the men (76.8%), although this difference was not statistically significant. Additionally, there was no significant correlation between gender and comfort with prolonged usage (*χ*^2^ = 0.338, df = 1, *p* = 0.561; Fisher's exact *p* = 0.628). Overall, the majority of the participants in both groups said they felt at ease using the chewable toothpaste tablet for extended periods of time (Table 5).

**Table 5:** Gender-wise distribution of participant-reported feedback.

Variable	Category	Male, *n* (%)	Female, *n* (%)	*p*-value
Experience	Excellent	11 (11.6%)	13 (12.9%)	0.306^a^
	Very good	52 (54.7%)	64 (63.4%)	
	Good	32 (33.7%)	24 (23.8%)	
Satisfaction	Satisfied	63 (66.3%)	65 (64.4%)	0.773^a^
	Extremely satisfied	32 (33.7%)	36 (35.6%)	
Side effects	No	95 (100%)	101 (100%)	—
Willingness to continue	Yes	73 (76.8%)	79 (78.2%)	0.818^a^
	No	22 (23.2%)	22 (21.8%)	
Comfort with extended use	Yes	68 (71.6%)	76 (75.2%)	0.561^a^
	No	27 (28.4%)	25 (24.8%)	

a Chi-square test.

**Figure 2:**
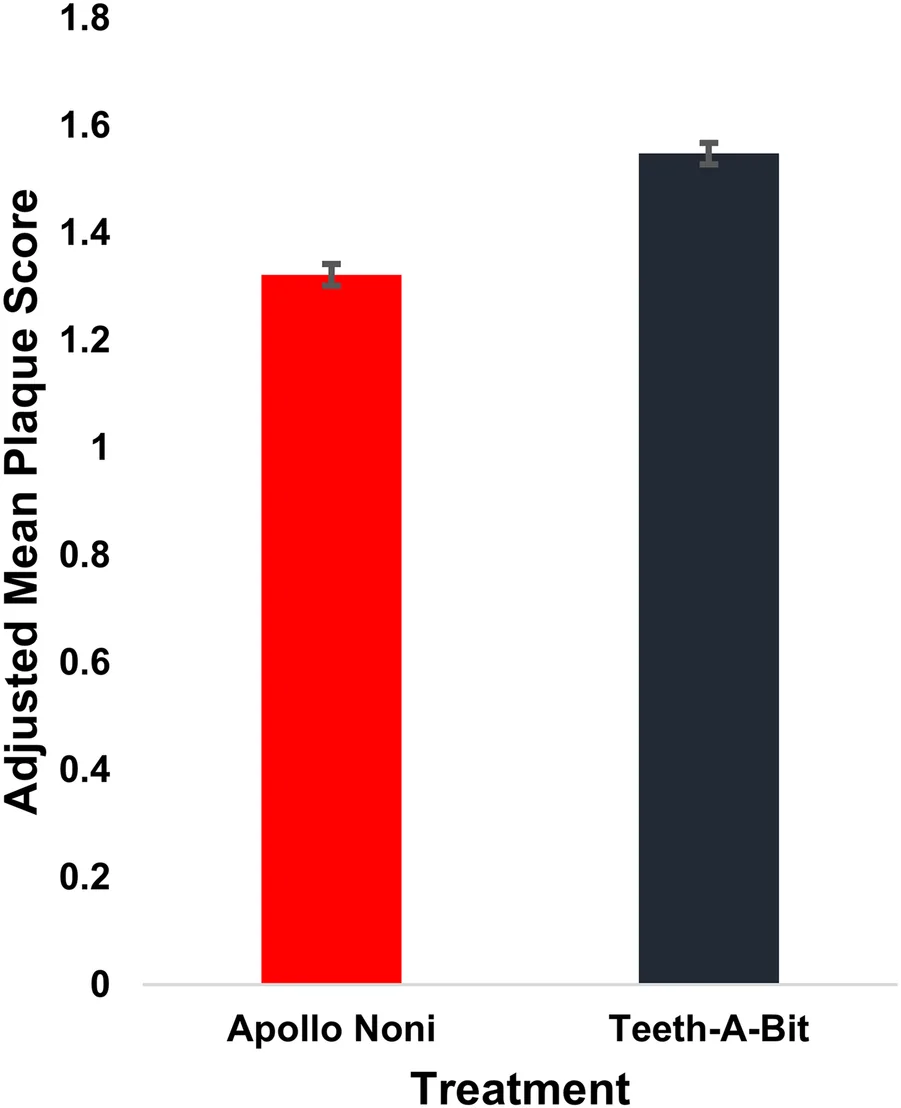
Adjusted mean plaque scores by treatment (REML model).

Adjusted marginal mean plaque scores were estimated using a linear mixed-effects model fitted with REML. The crossover model accounted for treatment, period, sequence, and baseline plaque score effects, with subject included as a random intercept. Apollo Noni demonstrated an adjusted mean plaque score of 1.322 (95% CI: 1.302–1.342), whereas Teeth-A-Bit demonstrated an adjusted mean plaque score of 1.548 (95% CI: 1.528–1.568). The error bars represent the 95% confidence intervals. A pairwise comparison revealed that Apollo Noni achieved significantly lower plaque scores than Teeth-A-Bit, with an adjusted mean difference of −0.226 (95% CI: −0.245 to −0.206; *p* < 0.001) (Figure 2).

## Discussion

The antiplaque effectiveness and user acceptability of two commercially available chewable toothpaste tablet formulations were assessed and compared among young people in this randomized double-blind crossover clinical experiment. The results showed that both formulations may reduce plaque buildup; however, Apollo Noni's antiplaque efficacy was noticeably higher than that of Teeth-A-Bit.

The primary etiological component responsible for the development and progression of periodontal disorders is still dental plaque biofilm (2–4). Therefore, the cornerstone of preventative oral healthcare is the efficient rupture of plaque biofilm by mechanical and chemical plaque control techniques (5). The idea that chewable toothpaste tablets could act as efficient substitute dentifrice delivery devices is supported by the decreases in plaque scores found in this study. The synergistic antibacterial and antibiofilm activity of Apollo Noni's herbal components, such as *S. persica*, clove, and cinnamon, may be the reason for its excellent antiplaque performance. *S. persica* contains bioactive compounds such as salvadorine, fluoride, silica, sulfur compounds, and tannins that exhibit antibacterial activity against periodontal pathogens and contribute to plaque inhibition (13–16, 23, 24, 26, 27, 36, 38). Clove and cinnamon additionally possess antimicrobial, anti-inflammatory, and antioxidant properties that may further contribute to inhibition of oral microbial colonization and biofilm disruption (11, 15).

The present findings are consistent with previous investigations evaluating herbal dentifrices and alternative oral hygiene products. Bhat et al. reported significant plaque reduction associated with propolis-containing herbal toothpaste in a crossover clinical trial (11). Similar antiplaque efficacy associated with herbal dentifrices containing meswak and tea tree oil has also been demonstrated previously (15). Ramli et al., in a systematic review and meta-analysis, further reported favorable plaque and gingival health outcomes associated with miswak-containing oral hygiene products (24). Recent clinical trials evaluating chewable toothpaste tablets have additionally demonstrated encouraging results comparable to conventional dentifrices (28–33).

The statistically significant treatment effect observed in our investigation confirms that Apollo Noni demonstrated superior plaque-removing efficacy compared with Teeth-A-Bit. Although the observed reduction in plaque scores was modest, it is clinically meaningful because even small reductions in plaque accumulation may improve oral hygiene when maintained consistently. Previous evidence has shown that dentifrices producing modest reductions in plaque regrowth can contribute to improved plaque control over time (36). Furthermore, the absence of a statistically significant sequence effect suggests minimal evidence of residual carryover and supports the methodological adequacy of the crossover design. Similar observations regarding limited carryover effects have previously been reported in crossover antiplaque dentifrice investigations employing short washout durations (20, 21, 37).

The analysis revealed no statistically significant period effect (*p* = 0.645), indicating that the treatment effects were not influenced by the order in which the interventions were administered. This observation may be the result of behavioral changes brought on by taking part in a clinical study, such as increased awareness of oral hygiene and brushing compliance in the early stages of the study. This may be because of the Hawthorne effect, which occurs when people change their behavior when they know they are being observed (39).

Participant acceptance is one of the most important factors that affects long-term compliance with dental health procedures. The majority of the study participants experienced high satisfaction, were willing to persevere with chewable toothpaste tablets, and were comfortable using them for lengthy periods of time. Most importantly, no unwanted effects were reported, indicating that the tolerance and safety profile of both formulations is satisfactory. Some methodological strengths of this study include the randomized double-blind crossover design, examiner calibration, uniform oral hygiene training, validated plaque assessment methodologies, and the strong statistical analysis (mixed-effects modeling). However, it 's important to acknowledge some limitations as well. First, participant acceptability was assessed using a study-specific self-reported exploratory questionnaire without formal validation or reliability testing that may be subject to response bias; the absence of a conventional fluoride toothpaste control group limited direct comparison with standard dentifrices; and the short duration of the intervention in the study did not allow for the assessment of gingival inflammation, caries prevention, or oral health outcomes in the long term. Second, a microbiological analysis of the composition of the plaque biofilm was not performed. Third, because there were different flavor attributes and flavor profiles across the chewable toothpaste tablet formulations, there was a potential for some participant unblinding, although double blinding was attempted with identical coded packaging. Furthermore, it is possible that the study itself affected the development of better oral hygiene behaviors (Hawthorne effect) (35). Finally, generalizability may be restricted since this was a single-center study. The long-term advantages of chewable toothpaste tablets for gingival health, oral microbiota modulation, caries prevention, and patient adherence in broader and more varied populations should be investigated and standardized, validated patient-reported outcome measures should be employed in future clinical trials.

## Conclusion

Both the chewable toothpaste tablets exhibited short-term efficacy in decreasing dental plaque accumulation within the restrictions of this randomized double-blind crossover clinical investigation. However, Apollo Noni demonstrated significantly greater antiplaque efficacy when compared with Teeth-A-Bit. The absence of significant sequence effects suggested minimal evidence of residual carryover and supported the methodological validity of the crossover design. Both chewable toothpaste tablets demonstrated favorable user acceptability and were well-tolerated without any adverse effects.

The findings of this study suggest that chewable toothpaste tablets may be an alternative to conventional dentifrices in preventive dentistry. However, further long-term investigations evaluating gingival health, microbiological outcomes, and caries prevention are recommended.

## Data Availability

The raw data supporting the conclusions of this article will be made available by the authors, without undue reservation.
