# Correction: Evaluation of the plaque removal efficacy of chewable toothpaste tablets – a randomized controlled crossover trial

**DOI:** 10.3389/froh.2026.1988531

**Published:** 2026-09-18

**Authors:** Harshit Srivastava, Kumar Gaurav Chhabra, Naganandini S., Pankaj Chaudhary, Shweta Dangi, Nimisha Srivastava

**Affiliations:** 1 Department of Public Health Dentistry, Nims Dental College and Hospital, Nims University Rajasthan, Jaipur, India; 2 Department of Oral Medicine and Radiology, Nims Dental College and Hospital, Nims University Rajasthan, Jaipur, India

**Keywords:** chewable toothpaste tablets, herbal dentifrice, plaque control, preventive dentistry, randomized crossover trial, sustainable oral healthcare

Affiliation Department of Public Health Dentistry, Nims Dental College and Hospital, Nims University Rajasthan, Jaipur, India was erroneously omitted for author Shweta Dangi. This affiliation has now been added for author Shweta Dangi.

Affiliation Department of Neurosurgery, National Institute of Mental Health and Neurosciences, Bengaluru, India was erroneously included in the paper. This affiliation has now been removed for author Shweta Dangi.

The original version of this article has been updated.

